# One Change, Many Benefits: A Glycine-Modified Bacteriochlorin with NIR Absorption and a Type I Photochemical Mechanism for Versatile Photodynamic Therapy

**DOI:** 10.3390/ijms252313132

**Published:** 2024-12-06

**Authors:** Mateusz Werłos, Agata Barzowska-Gogola, Barbara Pucelik, Paweł Repetowski, Marta Warszyńska, Janusz M. Dąbrowski

**Affiliations:** 1 Faculty of Chemistry, Jagiellonian University, 30-387 Kraków, Poland; matiwerner@gmail.com (M.W.); agata.barzowska@kit.lukasiewicz.gov.pl (A.B.-G.); barbara.pucelik@kit.lukasiewicz.gov.pl (B.P.); pawel.repetowski@doctoral.uj.edu.pl (P.R.); m.warszynska@doctoral.uj.edu.pl (M.W.); 2 Chemistry Department, Selvita, Podole 69, 30-394 Kraków, Poland; 3 Łukasiewicz Research Network, Kraków Institute of Technology, 30-418 Kraków, Poland; 4 Doctoral School of Exact and Natural Sciences, Jagiellonian University, 30-348 Kraków, Poland

**Keywords:** anticancer activity, bacteriochlorins, fluorescence imaging, photodynamic therapy (PDT), photosensitizers, porphyrins, reactive oxygen species (ROS), tumors

## Abstract

Difluorinated sulfonamide porphyrin (F_2_PGly) and bacteriochlorin (F_2_BGly), modified by glycine residues, were synthesized and evaluated for photodynamic therapy (PDT). F₂PGly exhibits superior stability and singlet oxygen generation efficiency but features a low-intensity band in the red range (λ_max_ = 639 nm). In contrast, F_2_BGly shows a favorable, red-shifted absorption spectrum (λ_max_ = 746 nm) that aligns well with phototherapeutic window, facilitating deeper tissue penetration. Moreover, it demonstrates reasonable photostability, necessary for the efficient generation of both singlet oxygen (type II) and oxygen-centered radicals (type I mechanism) which contributes to enhanced therapeutic efficacy. Importantly, the glycine modifications in F_2_BGly enhance its uptake in MCF-7 cells, known for their resistance to PDT due to efflux transport proteins like LAT1, showing great potential in the cancer cell-targeted PDT. The glycine groups potentially enable F_2_BGly to bypass these barriers, resulting in increased intracellular accumulation and more effective Reactive Oxygen Species (ROS) generation under illumination. In vivo studies indicated promising vascular-targeted PDT results, with real-time fluorescence imaging used to monitor photosensitizer distribution prior to irradiation. These findings suggest that F_2_BGly is a promising photosensitizer candidate with enhanced cancer cell selectivity and photodynamic efficiency, meriting further exploration in targeted PDT applications for multiple types of cancers.

## 1. Introduction

Photodynamic therapy (PDT) is a minimally invasive treatment approach approved for clinical use, particularly in oncology, where it combines a photosensitizer (PS), light, and molecular oxygen to generate reactive oxygen species (ROS), inducing oxidative stress at the site of illumination [1,2,3,4]. PDT works by harnessing these individually non-toxic components to initiate a series of biological reactions that target primary tumor sites and stimulate an antitumor immune response, potentially providing protection against metastasis [1,5]. The cornerstone of a successful treatment protocol involves two key steps, namely the administration of PS in the appropriate formulations and dosage [6], followed by the irradiation of the target tissue with the optimized light dose after a specific drug-to-light interval (DLI) to activate the PS [1,7]. To achieve maximum tissue penetration, PDT often uses photosensitizers that absorb in the near-infrared (NIR) range, within the phototherapeutic window (630–850 nm) [1,8], allowing for the more efficient interaction of photons with the tissue [9,10,11]. Upon light activation, PS reaches a first triplet excited state (T_1_) and can initiate two types of ROS-generating photochemical reactions. Type I involves electron transfer to form superoxide ions and hydroxyl radicals, while type II is an energy transfer reaction resulting in the generation of singlet oxygen [12,13,14]. Both pathways contribute to PDT’s therapeutic effects, including the direct destruction of cancer cells, blood vessel closure, and the stimulation of the host immune system [15]. Efficient PDT requires photosensitizers with redox properties that allow them to participate in both photochemical mechanisms, but at the same time are sufficiently photostable, to generate ROS with high efficiency [16,17,18,19,20].

Despite PDT’s potential, challenges remain, particularly with regard to PS specificity and stability. Many PSs lack tumor selectivity, which can lead to photodamage in healthy tissues [21]. Additionally, common tetrapyrrole-based PSs are prone to aggregation in a hydrophilic environment, which can reduce ROS generation efficiency [18,22]. Therefore, photosensitizers must not form aggregates in the tumor microenvironment (TME). To address these limitations, researchers have explored various structural modifications, including the addition of bulky substituents or functional groups, encapsulation within delivery systems, and assembly with nanostructured materials [17,23,24,25,26,27].

Moreover, the conjugation of PSs with biomolecules, such as antibodies, peptides, and small molecules, has emerged as a strategy for increasing tumor specificity [28,29,30]. These conjugates can selectively accumulate in tumors by targeting overexpressed receptors on cancer cells [31,32,33,34]. Peptides and amino acids are particularly advantageous as targeting ligands due to their biocompatibility, ease of chemical modification, and favorable pharmacological properties, making them suitable for developing sophisticated PDT formulations [35,36,37,38,39,40,41]. They also provide programmable structures, tunable self-assembly, and easy accessibility. Lysine, polylysine, and taurine derivatives are frequently reported in the literature as amino acid-based modifications [42,43]. However, designing advanced PS delivery systems with improved PDT efficacy using these simple components remains challenging [44]. Therefore, strategies involving the cooperative self-assembly of peptides and photosensitive drugs are crucial. Given their enhanced solubility and cell membrane interactions, amino acid-modified porphyrins may serve as strong PDT candidates [45,46,47].

In this study, we present novel difluorinated sulfonamide porphyrin and bacteriochlorin photosensitizers conjugated with glycine to improve amphiphilicity and cancer cells targeting through receptor-mediated endocytosis. A small modification—a reduction of two double bonds in the porphyrin structure (F_2_PGly)—leads to a compound with distinctly different properties (F_2_BGly), transforming its photophysical and photochemical behavior. This PS conjugate addresses key challenges in PDT, including enhanced NIR absorption, optimal stability, and diverse ROS generation, including superoxide ions and hydroxyl radicals. We characterize its spectroscopic and photochemical properties and evaluate its photodynamic effects both in vitro, using breast cancer (MCF7, MDA-MB-231) and colon carcinoma (CT26) cell lines, as well as in vivo, using a murine colon carcinoma in Balb/C mice. We optimized drug-to-light interval (DLI) for PDT by the in vivo fluorescence imaging of tumor-bearing mice. F_2_BGly possesses a shift in absorption to NIR, which increases light penetration through tissues and a switch from the type II photochemical mechanism typical for its porphyrin analog (F_2_PGly) to the type I mechanism, less dependent on the concentration of the surrounding molecular oxygen, which is particularly important in PDT in vivo. It has become apparent once again in the process of drug discovery that one seemingly small change in the chemical structure (four hydrogen atoms) can lead to a compound with new opportunities; in this case, a more versatile PS not only for cancer cell-targeted PDT but also vascular-targeted PDT.

## 2. Results

### 2.1. Synthesis of 5,10,15,20-Tetrakis[2,6-difluoro-3-(methoxycarbonylmethylsulfamido)-phenyl] Bacteriochlorin

**Figure 1:**
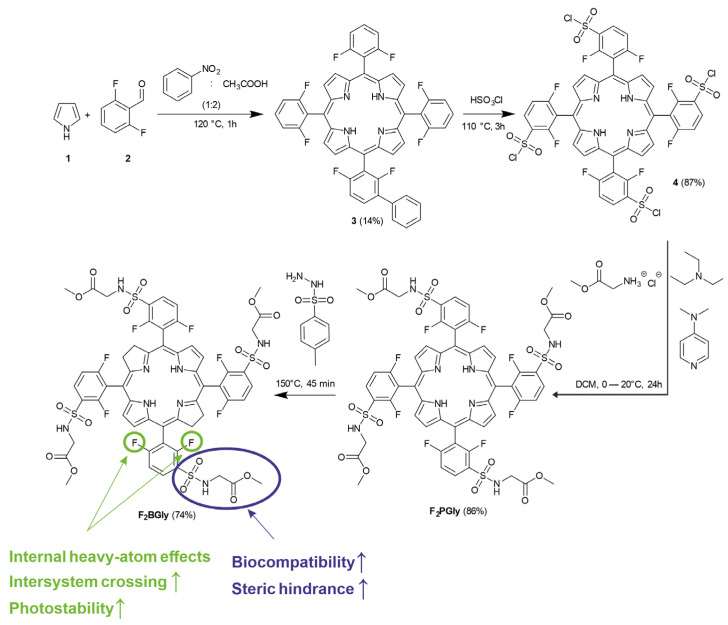
A synthetic scheme showing the preparation of porphyrin (F_2_PGly) and its subsequent reduction to bacteriochlorin (F_2_BGly). The incorporation of fluorine atoms enhances intersystem crossing via internal heavy atom effects, promoting an efficient transition to the triplet state. The glycine groups enhance the biocompatibility of the molecule and, through steric hindrance, contribute to its reasonable photostability, ensuring suitability for biological applications.

The synthesis of the bacteriochlorin used in the studies started with obtaining 5,10,15,20-tetrakis(2,6-difluorophenyl) porphyrin. 2,6-difluorobenzaldehyde and pyrrole were added to a mixture of acetic acid and nitrobenzene (Figure 1). In the next step, triethylamine and N,N-dimethyl-4-aminopyridine were added to glycine methyl ester hydrochloride in DCM. A solution of 5,10,15,20-tetrakis(2,6-difluoro-3-sulfonyl-phenyl) porphyrin was added dropwise to the mixture and stirred for 48 h under nitrogen. The reaction mixture was diluted with DCM, and washed with HCl, water, and brine. The solvent was removed under reduced pressure, and the product, F_2_PGly—5,10,15,20-tetrakis[2,6-difluoro-3-(methoxycarbonylmethylsulfamido)-phenyl] porphyrin, was purified using column chromatography.

The corresponding bacteriochlorin was synthesized through a diimide-mediated reduction in the porphyrin precursor. The resulting product, F_2_BGly (5,10,15,20-tetrakis[2,6-difluoro-3-(methoxycarbonylmethylsulfamido)-phenyl] bacteriochlorin), was purified using column chromatography (Figure 1).

### 2.2. Optical and Photophysical Properties

**Figure 2:**
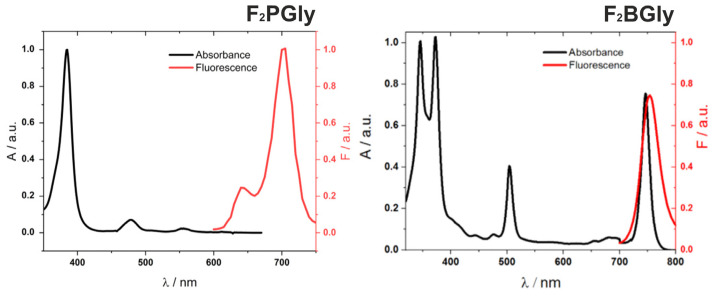
Normalized electronic absorption and emission spectra of porphyrin and bacteriochlorin in ethanol. Fluorescence spectra were recorded upon excitation at 505 nm for F_2_BGly and at 410 nm for F_2_PGly.

The electronic absorption spectrum of the synthesized porphyrin is presented in Figure 2 and exhibits the typical features of this class of macrocycles, with the longest wavelength absorption observed at 639 nm and a molar absorption coefficient (ε) of 7980 M^−1^ cm^−1^ for this band (Table 1). Reducing the two pyrrole rings on opposite sides of the macrocycle not only enhances electronic absorption at longer wavelengths, as evidenced by the appearance of the Q_y_-band at 746 nm, but also significantly impacts the electronic properties of the macrocycle. This reduction leads to a narrower energy gap and an increased HOMO energy level, which typically compromises stability [48]. However, in the case of F_2_BGly, the introduction of electron-withdrawing fluorine atoms in the ortho-positions and steric hindrance provided by the glycine group partially counteract these effects. Compared to other photosensitizers, such as mTHPBC (5,10,15,20-tetrakis(m-hydroxyphenyl)bacteriochlorin), for which photobleaching rates are documented in various experimental setups, F_2_BGly demonstrates superior stability under comparable irradiation conditions [49,50]. The photodegradation results of F_2_BGly, along with those of another bacteriochlorin (F_2_BOH) for comparison, are presented in Figure S1. Upon increasing the light dose, F_2_BGly exhibits a negligible decrease in absorption, indicating its enhanced photostability. In contrast, the F_2_BOH compound undergoes significant photodegradation, with approximately 50% of its initial absorption lost after exposure to a light dose of 5 J/cm^2^.

The F_2_PGly fluorescence spectrum shows two emission bands at 642 nm and 705 nm, with a fluorescence quantum yield of Φ_F_ = 0.037. In comparison, F_2_BGly demonstrates a red-shifted emission spectrum (λ_max_ = 764 nm) and enhanced fluorescence quantum yield (Φ_F_ = 0.096). The fluorescence lifetime, determined using the single-photon counting method, is measured as 4 ns for F_2_BGly (Figure S2) and is comparable to that of F_2_BMet. This relatively short fluorescence lifetime can be attributed to the presence of eight fluorine atoms localized in the phenyl groups placed in the meso-position of the macrocycle.

Additionally, logP_OW_ were determined for F_2_BGly, the corresponding porphyrin, and reference bacteriochlorin, and are collected in Table 1 together with the optical and photophysical properties.

### 2.3. ROS Detection by Fluorescent Probes

PDT can be classified into type I and type II reactions, differentiated by the photophysical and photochemical mechanisms. The type I/II ratio depends on the properties of PS, the substrate molecules, and the surrounding environment [12]. The ability of F_2_BGly to produce various ROS upon illumination was monitored using the fluorescent probes DHE, SOSG, APF, and HPF. Singlet Oxygen Sensor Green (SOSG) is highly selective for singlet oxygen, with minimal sensitivity to superoxide ions and hydroxyl radicals (OH). In contrast, 3′-(p-aminophenyl) fluorescein (APF) and 3′-(p-hydroxyphenyl) fluorescein (HPF) exhibit high selectivity for ^•^OH, producing fluorescein upon reaction with these radicals. Additionally, dihydroethidium (DHE) serves as a reliable probe for detecting superoxide anion radicals (O_2_^•^⁻).

**Figure 3:**
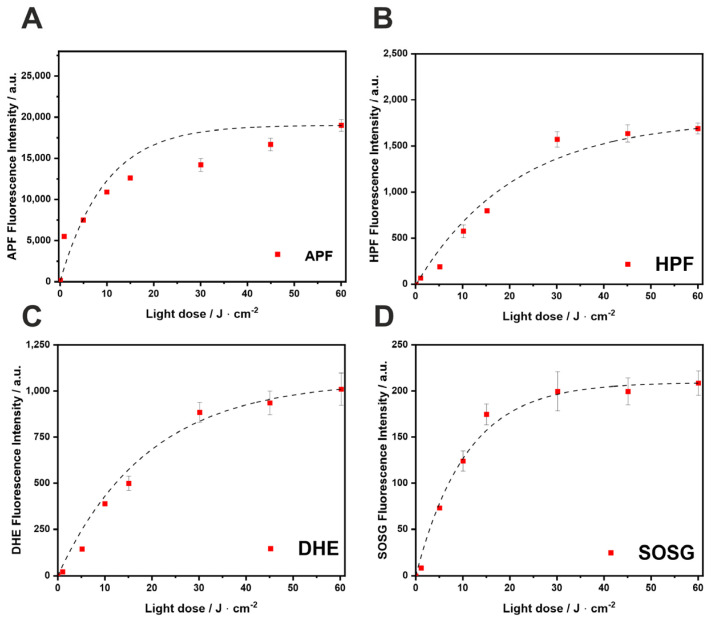
The generation of ROS in F_2_BGly solution (in phosphate-buffered saline (PBS) and dimethyl sulfoxide (DMSO) not exceeding 0.5%) assessed by fluorescence probes selective towards (**A**) overall radical species (APF); (**B**) hydroxyl radicals (HPF); (**C**) superoxide radical anion (DHE); and (**D**) singlet oxygen (SOSG). The bacteriochlorin concentration was 5 μM in all samples, which was irradiated with a 735 ± 20 nm LED light. Data are presented as mean ± the standard error of the mean (SEM) (N = 12).

As shown in Figure 3, the investigated bacteriochlorin activates probes specific for singlet oxygen and for oxygen-centered radicals, indicating that at therapeutic doses, both type I and type II mechanisms are involved. In contrast, the irradiation of F_2_PGly with light at 635 ± 20 nm predominantly generates singlet oxygen (Figure S3). Thus, the reduction of double bonds in the macrocycle shifts the photochemical mechanism from type II to type I. This modification leads to a higher generation of ROS, such as superoxide ion and hydroxyl radicals, in addition to singlet oxygen.

For several bacteriochlorins reported in the literature, the type I photochemical pathway involving photoinduced electron transfer is dominant [17]. Photosensitizers favoring type I processes typically exhibit good electron-donating properties and low oxidation potentials. The studied bacteriochlorin also generates superoxide ions, as detected with DHE (Figure 3C), likely acting as the primary ROS in these photoredox reactions. The balance between type I (hydroxyl radicals) and type II (singlet oxygen) mechanisms is critical for PDT efficacy. Hydroxyl radicals, being the most cytotoxic ROS, serve as the powerful tumor-damaging agents in anticancer PDT. Tookad^®^ Soluble [51], another bacteriochlorin derivative, operates exclusively via type I photochemistry in aqueous media [52,53]. Upon light activation, this PS generates both superoxide and hydroxyl radicals. The interaction between superoxide and nitric oxide in tumor blood vessels has been proposed as a key factor in the antivascular effects of Tookad^®^-V-PDT [12,53]. The tendency of bacteriochlorins to generate ROS through a type I mechanism is attributed to the formation of charge-transfer complexes with molecular oxygen [1,12,54].

### 2.4. Biological Studies In Vitro

**Figure 4:**
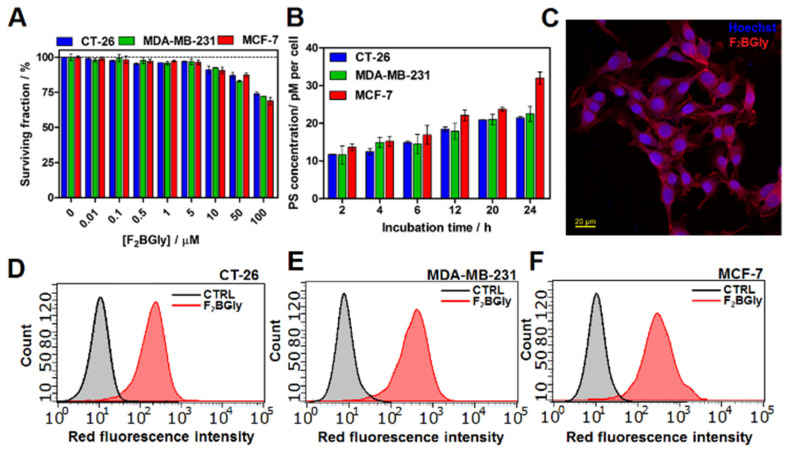
(**A**) The changes in metabolic activity induced by F_2_BGly in CT-26, MDA-MB-231, and MCF-7 cell lines. (**B**) The time-dependent uptake of F_2_BGly prepared in PBS/0.5% DMSO measured by fluorescence intensity. The results represent the mean of at least three independent experiments ± SEM. (**C**) Fluorescence microscopy images of MCF-7 cells showing accumulated F_2_BGly, visible as characteristic red fluorescence. (**D**) Flow cytometry analysis of F_2_BGly uptake in CT-26; (**E**) MDA-MB-231; (**F**) MCF-7 cells.

We next examined the cellular uptake, mitochondrial metabolic activity, and photodynamic activity of F_2_BGly on CT26, MCF-7, and MDA-MB-231. The mitochondrial activity of F_2_BGly and F_2_PGly was assessed using the MTT assay (Figure 4A, Figure S4). It is important to note that the MTT assay does not directly measure the surviving fraction of cells but rather evaluates mitochondrial metabolic activity, specifically the activity of NAD(P)H-dependent oxidoreductase enzymes. The results can be interpreted as an indicator of the relative mitochondrial function of cells in each well, providing an indirect assessment of cell viability. The data indicate that the investigated PS did not significantly affect mitochondrial activity across a wide range of concentrations (up to 50 µM). A reduction in mitochondrial activity of approximately 25% was observed at a concentration of 100 µM, which was consistent with the results obtained for F_2_PGly. However, a reliable assessment of dark toxicity would ideally require clonogenic assays to confirm the impact on the surviving fraction of cells.

For effective anticancer activity, particularly in cellular-targeted PDT protocols, photosensitizers need to be internalized by cells. Therefore, preliminary evaluations of uptake were performed using cellular extracts, flow cytometry, and fluorescence microscopy. As shown in Figure 4B, the optimal incubation time for efficient accumulation was determined for each cell line tested. In all cases, maximal uptake of the PS occurred after 24 h of incubation. A quantitative analysis of cell lysates indicated that MCF-7 cells accumulated relatively more F_2_BGly compared to CT26 and MDA-MB-231 cells (Figure 4B). Additionally, the time-dependent uptake of F_2_PGly in CT26 cells showed a similar pattern to F_2_BGly, with peak accumulation observed after 18 h of incubation (Figure S5). This uptake was further confirmed by fluorescence microscopy (Figure 4C) and flow cytometry analysis, which demonstrated consistent accumulation across the treated cancer cell populations (Figure 4D–F).

**Figure 5:**
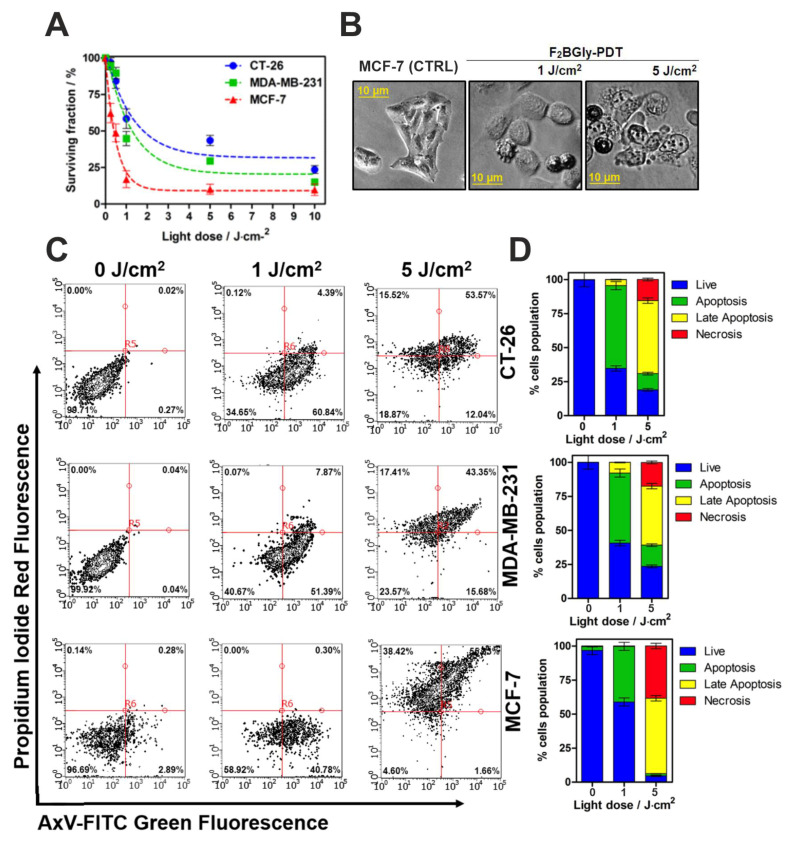
(**A**) The photodynamic effect of F_2_BGly evaluated on CT-26, MDA-MB-231 and MCF-7 cell lines. (**B**) The morphological changes in MCF-7 cells observed before and after PDT with varying light doses (from left to right: control cells, cells treated with light doses of 1 J/cm^2^ and 5 J/cm^2^, respectively). (**C**) Flow cytometry analysis of CT-26, MDA-MB-231 and MCF-7 cells incubated with 5 µM F_2_BGly for 24 h, followed by treatment without light (0 J/cm^2^) or with light doses of 1 J/cm^2^ and 5 J/cm^2^. Cells were stained with Annexin V and PI to distinguish live cells (lower left quadrant), early apoptotic cells (lower right quadrant), late apoptotic cells (upper right quadrant), and necrotic cells (upper left quadrant). (**D**) The quantification of cell-death pathways (apoptosis and necrosis) at different light doses during PDT.

The studied bacteriochlorin derivative demonstrates high phototoxic effectiveness against MCF-7, MDA-MB-231, and CT26 cells upon NIR irradiation with a relatively low-light dose (up to 10 J/cm^2^). F_2_BGly exhibited light-dose-dependent phototoxicity in all tested cancer cell lines (Figure 5A). The photodynamic effect was most pronounced in MCF-7 cells compared to the other cell lines. At a light dose of 10 J/cm^2^, F_2_BGly-mediated photodynamic activity resulted in the nearly complete destruction of MCF-7 cells, reducing viability to 15% and 25% in MDA-MB-231 and CT26 cells, respectively. Representative images of treated MCF-7 cells are presented in Figure 5B.

To confirm F_2_BGly’s ability to induce cell death in cancer cells, Annexin V/PI staining was performed. Additionally, Figure S6 illustrates the photodynamic effect of the porphyrin F_2_PGly on CT26 cells, showing that even after irradiation at a higher dose of 20 J/cm^2^, more than 50% of the cells remained viable. As the murine colon tumor cell line (CT26) exhibited the highest resistance to F_2_BGly PDT (achieving a 30% surviving fraction after the highest irradiation dose), F_2_PGly proved to be even less effective in this model and was not tested on other cell lines.

The percentage of apoptotic (early and late) and necrotic cells was determined using flow cytometry following photodynamic treatment with two light doses: 1 and 5 J/cm^2^, respectively (Figure 5C). Figure 5D presents the average percentages of apoptosis observed after respective treatments in CT26, MDA-MB-231, and MCF-7 cells. The results indicate that irradiation with 1 J/cm^2^ induces apoptosis as the predominant cell-death pathway, particularly in CT26 cells. However, at a higher light dose (5 J/cm^2^), the proportion of necrotic cells increases, especially in the MCF-7 cell line.

**Figure 6:**
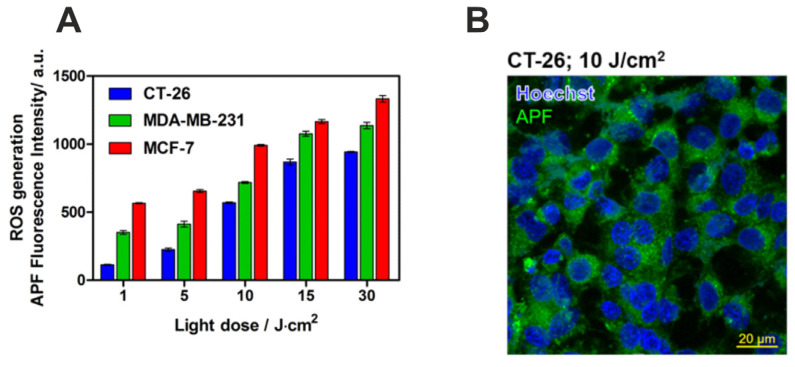
(**A**) The generation of ROS assessed by the APF in CT26, MDA-MB-231, and MCF-7 cell lines. (**B**) Fluorescence confocal imaging of CT26 to show the intracellular accumulation of PS in cancer cells after 24 h incubation with F_2_BGly, visualized with Hoechst33342.

To confirm that phototoxicity is linked to increased intracellular ROS levels, the APF probe was used to measure ROS generation. The production of ROS by F_2_BGly accumulated in CT26 murine colon carcinoma and human breast cancer cell lines (MCF-7 and MDA-MB-231) was validated by increased fluorescence intensity following irradiation with light doses ranging from 1 to 30 J/cm^2^. As expected, the strongest fluorescence signal was observed in MCF-7 cells, consistent with the highest PS accumulation after 24 h of incubation (Figure 6A). These findings indicate that F_2_BGly effectively generates ROS in vitro, facilitating both type I and type II photoreactions.

### 2.5. Fluorescence Imaging and Photodynamic Therapy In Vivo

**Figure 7:**
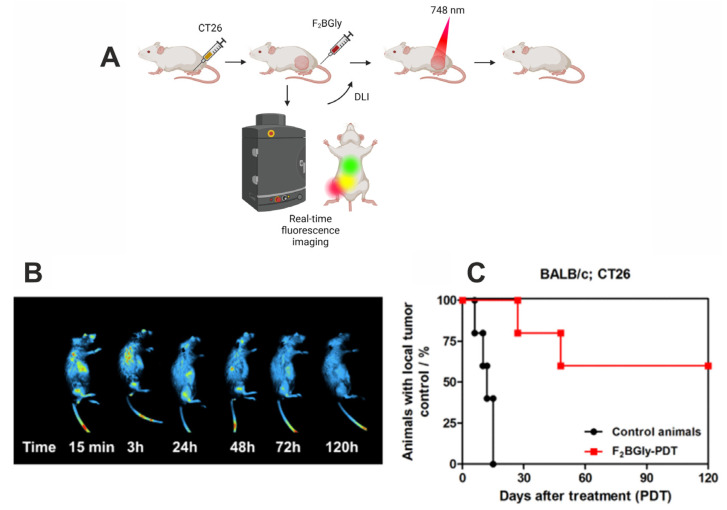
(**A**) A schematic representation of the in vivo photodynamic effect evaluation using real-time fluorescence imaging to determine the drug-to-light interval (DLI). (**B**) Whole-body fluorescence imaging of BALB/c mice bearing a CT-26 tumor in the right leg, collected after 15 min, 3, 24, 48, 72 and 120 h after the intravenous administration of 1.5 mg/kg of F_2_BGly with excitation at 680 nm (excitation range: 650–690 nm) and fluorescence emission at 750 nm (detection range: 710–755 nm). (**C**) The antitumor efficacy of F_2_BGly against CT26 tumors in BALB/c mice model at DLI = 15 min.

In vivo fluorescence imaging was used to follow the accumulation of F_2_BGly in tumor tissues and to determine the drug-to-light interval for the optimal PDT treatment (Figure 7A). Imaging was performed using laser excitation at 680 nm and fluorescence signal detection in the range of 740–780 nm. Despite the lower photodynamic effect observed in vitro, the CT26 cell line was chosen due to its ease of maintenance and the lack of a need for specialized in vivo models, such as the MCF-7 xenograft nude mice model. Figure 7B presents representative images of mice with CT26 tumors in the right leg following an intravenous injection of F_2_BGly. The fluorescence images reveal that F_2_BGly accumulates rapidly in the tumor immediately after injection, with the fluorescence signal gradually decreasing until 120 h post injection, at which point no emission around the tumor is detected. Shortly after injection, the photosensitizer is primarily localized in the vasculature and tumor microenvironment, without significant penetration into cancer cells. This suggests that the optimal protocol for treating CT26 tumors is the vascular protocol (V-PDT), consistent with in vitro findings, which show that F_2_BGly is taken up more slowly by CT26 cells compared to MCF-7 cells.

Our assumptions regarding the localization and application of the V-PDT protocol were validated by in vivo PDT treatment. CT26 tumors in BALB/c mice were irradiated 15 min after PS administration, resulting in a long-term survival rate of 60% for treated animals (Figure 7C). Furthermore, this high survival rate persisted for 4 months post treatment, highlighting the effectiveness of the applied photosensitizer and treatment regimen. Notably, we present the long-term outcomes of F_2_BGly-mediated V-PDT, whereas many studies focus only on short-term effects observed within 20–30 days post treatment [55,56,57]. Limiting survival analyses to such short timeframes can obscure the true efficacy of the therapy, as they fail to capture potential long-term benefits. By extending the observation period, our results provide a more comprehensive evaluation of the treatment’s effectiveness.

## 3. Discussion

The synthesized glycine-modified difluorinated sulfonamide bacteriochlorin (F_2_BGly) exhibits a promising characteristic as a photosensitizer (PS) for the V-PDT of colon tumors. This novel bacteriochlorin was derived from its precursor porphyrin, F_2_PGly. A relatively simple structural modification, involving the reduction of two pyrrole units, significantly enhanced F_2_BGly’s absorption in the NIR region. This adjustment aligns the compound more effectively with the phototherapeutic window, facilitating deeper tissue penetration and potentially improving therapeutic efficacy in solid tumors. Remarkably, despite the notable decrease in the energy of the S_0_-S_1_ transition, the stability of the compound remains nearly uncompromised. F_2_BGly demonstrates a greater photostability compared to the sulfonated bacteriochlorin (F_2_BOH), as detailed in the Supplementary Materials (Figure S1), and comparable photostability to redaporfin (F_2_BMet) [6,58,59,60,61]. This is due to the steric hindrance caused by glycine residues and the electron-withdrawing effect of fluorine atoms.

The PDT mechanism of action involves the generation of ROS, critical for inducing cytotoxicity in cancer cells. Our study indicates that F_2_BGly favors a type I photochemical pathway, generating hydroxyl radicals, alongside the type II pathway producing singlet oxygen. This dual ROS generation strategy enhances its efficacy, as hydroxyl radicals, being more reactive, contribute significantly to the direct cytotoxic effects on tumor vasculature and cells. This contrasts with F_2_PGly, which primarily generates singlet oxygen, potentially limiting its photodynamic effect.

Furthermore, F_2_BGly demonstrates accumulation in various cancer cells, particularly MCF-7, as evidenced by fluorescence confocal microscopy, flow cytometry, and increased intracellular ROS levels post irradiation. This suggests that the glycine modification may enhance tumor cell uptake or retention. As reported in the literature, breast cancer cell lines may overexpress the breast cancer resistance protein (BCRP) on their surface [62,63]. Therefore, BCRP can remove macrocyclic compounds such as pheophorbide a, protoporhyrin IX (PpIX), chlorin e6, or porphyrins, making BCRP-positive cancers a difficult target for PDT [64,65]. Inspired by the approach used by Pocasap et al., in which chlorambucil was conjugated with tyrosine to enhance cellular uptake via LAT1 transporters, we designed the modified F_2_BGly photosensitizer with glycine residues [66]. MCF-7 cells also express P-glycoprotein, which contributes to their strong resistance to PDT by expelling photosensitizers [67,68,69,70]. However, the addition of glycine-containing groups to F_2_BGly may facilitate more effective transport into cells and improved retention, allowing enhanced ROS generation upon irradiation.

This mechanism aligns with findings in the referenced study, where the tyrosine–chlorambucil conjugate utilized LAT1-mediated transport to achieve higher intracellular drug concentrations in MCF-7 cells, resulting in a stronger cytotoxic effect [71]. For F_2_BGly, the glycine residues appear to play a similar role, potentially facilitating selective membrane transport via amino acid transport proteins and thereby increasing compound concentration within cancer cells. This is particularly crucial for MCF-7 cells, which are typically challenging to treat with PDT due to drug efflux mechanisms. Our results suggest that attaching amino acids like glycine to the PS structure may overcome these barriers, enhancing intracellular retention and cytotoxicity.

The in vitro findings for F_2_BGly, particularly its enhanced efficacy against MCF-7 cells compared to CT26 cells, may appear contradictory to its in vivo performance. However, in vitro studies predict only cellular effects, while the results do not necessarily translate directly to in vivo findings. Based solely on in vitro experiments, one might conclude that F_2_BGly is not ideal for the PDT of colon tumors, and the CT26 cell line could be excluded from further research. However, in vivo fluorescence imaging demonstrated a rapid accumulation of F_2_BGly in tumor vasculature within 15 min of administration, supporting its suitability for vascular-targeted PDT. An in vivo PDT evaluation confirmed this assumption, with CT26 tumor-bearing mice showing a long-term survival rate of 60%, extending to 4 months post treatment. The efficacy of F_2_BGly against MCF-7 cancer cells will continue to be explored, as in vitro experiments suggest strong potential for C-PDT. Future studies will establish the photosensitizer’s localization in animal models using real-time fluorescence imaging to determine the optimal drug-to-light interval. Following DLI evaluation, PDT studies on MCF-7 tumor-bearing mice for C-PDT will be conducted.

Our work also demonstrates that minor structural modifications to the tetrapyrrolic ring—specifically, the reduction of double bonds—can significantly enhance both the photochemical and biological activities of the compound. This adjustment shifts absorption into the NIR region, making the compound more suitable for PDT, while also altering the photochemical pathway from type II (singlet oxygen-dominated) to type I (radical species-dominated). The increased generation of type I photochemical reaction products renders glycine-modified bacteriochlorin particularly effective for treating hypoxic tumors. Structural modifications that improve selective transport and accumulation in cancer cells play a crucial role in developing more effective photosensitizers. Compared to its porphyrin analog, F_2_BGly exhibited a stronger photodynamic effect than F_2_PGly against the CT26 cell line and demonstrated high efficacy against MCF-7 and MDA-MB-231 cells. The addition of glycine substituents not only enhances intracellular transport and modifies ROS production but also enables a multi-targeted approach for in vivo therapy.

Most photosensitizers currently in clinical use have defined DLIs; for instance, Tookad^®^ and Tookad^®^ Soluble are employed in V-PDT with DLIs of 6 and 10 min, respectively, while 5-ALA (a PpIX precursor) is used topically with a 3 h DLI, and Photofrin requires a 24 h DLI [1,72,73,74,75,76,77,78,79]. Here, we demonstrate that simple modifications to the tetrapyrrolic ring—glycine addition and double bond reduction—can expand PDT applications across various cancers. In vitro results revealed strong efficacy against the MCF-7 breast cancer model, indicating potential for C-PDT. Furthermore, in vivo fluorescence imaging of CT26 tumor-bearing mice showed significant photosensitizer accumulation in tumor vasculature within 15 min post administration, confirming its potential for V-PDT. This was corroborated in vivo, where PDT treatment achieved a 60% long-term survival rate extending over 4 months. These results underscore the broad therapeutic potential of F_2_BGly and highlight how a single structural modification can provide multiple clinical benefits.

## 4. Materials and Methods

All reagents and solvents used in the experiments were obtained from commercial supplies and did not require additional purification. SiliaFlash P60 silica gel (SiliCycle, Québec, QC, Canada), was used for product purification via column chromatography, while TLC was performed on aluminum plates coated with silica gel 60 F254 (Merck, Rahway, NJ, USA). Chromatogram analysis on the plates was conducted using a UV lamp (254 nm) and ceromolybdenum-based stain. The NMR spectra were acquired at room temperature on a 300 MHz spectrometer (Brucker, Karlsruhe, Germany). Samples for NMR were prepared with approximately 15 mg of the compound in CDCl_3_ or DMSO.

### 4.1. Synthesis of 5,10,15,20-Tetrakis[2,6-difluoro-3-(methoxycarbonylmethylsulfamido)-phenyl] Bacteriochlorin

The synthesis began with 5,10,15,20-tetrakis(2,6-difluorophenyl)porphyrin as a precursor. This compound was synthesized by heating a mixture of 2,6-difluorobenzaldehyde (43 mM), pyrrole (3 mL, 43 mM), acetic acid (140 mL, 2.45 M), and nitrobenzene (70 mL, 0.68 M) at 120 °C for 1 h. Once cooled, 50 mL of methanol was added, and the product was purified using column chromatography. Next, the compound was reacted with chlorosulfonic acid (10 mL, 150 mM) at 110 °C for 3 h, then cooled to −20 °C before the addition of dichloromethane (200 mL). The solution was washed sequentially with water, 10% sodium bicarbonate solution, and dried over anhydrous magnesium sulfate. Column chromatography with dichloromethane as the eluent yielded 5,10,15,20-tetrakis(2,6-difluoro-3-sulfonylphenyl)porphyrin. Subsequently, glycine methyl ester hydrochloride (4 mM), triethylamine (8 mM), and a catalytic amount of N, N-dimethyl-4-aminopyridine in DCM were mixed, and a solution of the sulfonyl–porphyrin was added dropwise. The reaction proceeded under nitrogen for 48 h. After adding 30 mL of DCM to dilute the reaction mixture, it was sequentially washed with 1 M HCl, water, and brine. Solvent removal under reduced pressure and column chromatography produced F_2_PGly—5,10,15,20-tetrakis[2,6-difluoro-3-(methoxycarbonylmethylsulfamido)phenyl]porphyrin. The conversion to F_2_BGly (bacteriochlorin) involved mixing F_2_PGly (0.05 mM) with p-toluenesulfonic acid hydrazide (1:40 molar ratio) at 150 °C for 45 min. The product was then cooled and purified by column chromatography to yield F_2_BGly, confirmed via ^1^H NMR, elemental analysis, and electronic absorption spectroscopy.

5,10,15,20-tetrakis(2,6-difluorophenyl) porphyrin, yield = 14%; δH, ppm (300 MHz, CDCl_3_) 8.83 (s, 8H, β-H); 7.75 (m, 4H, Ph-H); 7.35 (m, 8H, Ph-H); −2.79 (s, 2H-NH); UV-Vis spectrum (THF); nm: 412; 506; 538; 584; 654; C_44_H_22_F_8_N_4_: calculated C 69.66, H 2.92, F 20.03, N 7.38; found C 69.80, H 2.98, N 7.26.

5,10,15,20-tetrakis(2,6-difluoro-3-sulfonyl-phenyl) porphyrin, yield = 87%; δH, ppm (300 MHz, CDCl_3_) 8.85 (s, 8H, β-H); 8.52 (m, 4H, Ph-H); 7.61 (m, 4H, Ph-H); −2.85 (s, 2H-NH).

5,10,15,20-tetrakis[2,6-difluoro-3-(methoxycarbonylmethylsulfamido)phenyl] porphyrin, yield = 86%; δH, ppm (300 MHz, DMSO-d_6_) 9.14 (s, 8H, β-H); 8.69 (s, 4H,-NH); 8.33 (m, 4H, Ph-H); 7.79 (m, 4H, Ph-H); 3.96 (s, 8H, -CH_2_-); 3.53 (s, 12H, -CH_3_); −3.13 (s, 2H, -NH); C_56_H_42_F_8_N_8_O_16_S_4_: calculated C 49.34, H 3.11, F 11.15, N 8.22, O 18.78, S 9.41; found C 49.61, H 3.01, N 7.82, S 9.71.

5,10,15,20-tetrakis[2,6-difluoro-3-(methoxycarbonylmethylsulfamido)-phenyl] bacteriochlorin, yield = 74%; δH, ppm (300 MHz, DMSO-d_6_) 8.56 (s, 4H, -NH-); 8.24 (s, 4H, β-H); 8.14 (m, 4H, Ph-H); 4.03 (s, 8H, β-H); 3.90 (s, 8H, -CH_2_-); 3.46 (s, 12H, -CH_3_); −1.46 (s, 2H, -NH).

### 4.2. Optical Properties

The electronic absorption spectra were recorded using a Shimadzu 3600 UV-Vis-NIR spectrometer (Kyoto, Japan). The photosensitizers solutions were prepared in EtOH. The measurement was carried out in a quartz cuvette with an optical path of l = 1 cm, in the range λ = 320–800 nm. The experiment was performed at room temperature, with limited light. Using measured absorbance for various concentrations of bacteriochlorin in EtOH, the molar absorption coefficients were determined from Beer’s law. Fluorescence emission spectra were recorded from 600 nm to 700 nm with excitation at the Soret band. Fluorescence spectra were measured using a Shimadzu RF-6000 Spectrofluorophotometer (Kyoto, Japan).

### 4.3. Fluorescence Quantum Yield Measurements

Fluorescence quantum yields (Φ_F_) were determined by comparing the integrated areas under each emission spectrum with the reference value for redaporfin (Φ_F_ = 0.138 in ethanol), as described by Equation (1).

(1) $$\Phi_{F}=\Phi_{Fref\;}\times \frac{F_{sample}}{1-10^{-Abs_{sample}}}\times \frac{1-10^{-Abs_{ref}}}{F_{ref}}\times \frac{{\eta^{2}}_{sample}}{{\eta^{2}}_{ref}}$$

where

*F_sample_* stands for the integration area under the emission spectrum, *Abs* represents the absorbance at the excitation wavelength, while *η* corresponds to the refractive index of the solvent used.

### 4.4. LogP Determination

The octanol/PBS partition coefficient was determined using the shaking method. Bacteriochlorin was dissolved in octanol, sonicated, and PBS was added. After vortex mixing and centrifugation at 3700 rpm, fluorescence spectra were recorded to calculate concentrations via a calibration curve.

### 4.5. Detection of Reactive Oxygen Species (ROS) with Fluorescent Probes

The 3′-p-(aminophenyl)fluorescein (APF) and hydroxyphenyl fluorescein (HPF) are selective probes for hydroxyl radicals. Singlet Oxygen Sensor Green^®^ (SOSG) is a specific probe for singlet oxygen, and dihydroethidium (DHE) is a probe for the identification of the superoxide ion. These probes were used to detect ROS following the illumination of the PS. The PS solutions were diluted to a final concentration of 10 μM in each well, and a fluorescent probe was subsequently added to reach a final concentration of 15 μM per well. The solutions were then exposed to 400 ± 20 nm LED light for varying doses. Fluorescence intensity was measured using a Tecan Infinite M200 Reader (Männedorf, Switzerland) both immediately before and after illumination, utilizing the relevant excitation and emission parameters.

### 4.6. Cell Cultures

CT-26 (murine colorectal carcinoma cell line), MDA-MB-231 (epithelial, human breast cancer), and MCF-7 (human breast cancer cell line with estrogen, progesterone, and glucocorticoid receptors) cells were grown in RPMI 1640 with the addition of 10% FBS and supplemented by antibiotics (100 IU mL^−1^ penicillin and 100 mg·mL^−1^ streptomycin). The cells were maintained under fully humidified conditions at 37 °C with 5% CO₂. All experiments were conducted with cells in the logarithmic growth phase. Media were replaced every 2 days and cells were subcultured using 0.25% trypsin EDTA.

### 4.7. Cellular Uptake

CT-26, MDA-MB-231, and MCF-7 cells were seeded on a 96-well microplate (10^4^ per well). After 24 h, the cells were incubated with 5 µM F_2_BGly for time intervals from 2 h up to 24 h. F_2_BGly solutions were prepared by diluting its stock solution in DMSO with the culture medium to the desired final concentration (5 μM). The highest concentration of DMSO in the cell-growth medium did not exceed 0.5%. Following incubation, the cells were washed twice with PBS and solubilized in 30 μL of Triton X-100 along with 70 μL of a DMSO/ethanol solution (1:3). The retention of cell-associated F_2_BGly was detected by fluorescence (λ_exc_ = 505, λ_em_ = 750 nm) with the microplate reader (Tecan Infinite M200 Reader).

### 4.8. Detection of Reactive Oxygen Species In Vitro

The APF fluorescent probe was employed for the detection of ROS formation during irradiation. CT26, MDA-MB-231, and MCF-7 cells were incubated with 5 μM F_2_BGly for 24 h. Two hours before the end of incubation, APF (15 μM) was added to the solution. The cells were then washed with PBS and exposed to irradiation by a 735 ± 20 nm light source for various time intervals. The APF fluorescence signals (λ_exc_ = 488 nm, λ_em_ = 515 nm) were determined using a microplate reader (Tecan Infinite M200 Reader) immediately before and after illumination.

### 4.9. Cell Metabolic Activity Assay

The MTT (3-(4,5-dimethylthiazol-2-yl)2,5-diphenyl tetrazolium bromide) was used to indirectly quantify cell survival upon the treatment with PSs and PSs along with light illumination. After cell attachment to the 96-well plate, F_2_BGly in a growth medium at concentrations from 0 to 100 μM was added to the cells. The cells were incubated for 24 h in the dark. Next, the F_2_BGly solution from each well was removed, cells were washed in PBS, fresh culture medium supplemented with FBS and antibiotics was added to each well, and cells were returned to the incubator for 24 h. MTT, dissolved in PBS at a proportion of 10% of the final solution, was added to each well and the microplates were further incubated for ca. 3 h. For the MTT assay, the medium was discarded, and 100 μL of a DMSO/methanol mixture (1:1) was added to the cultures and thoroughly mixed to dissolve the dark-blue formazan crystals. Formazan was quantified using an automated microplate reader (Tecan Infinite M200 Reader) by measuring absorbance at a test wavelength of 565 nm.

### 4.10. Photodynamic Effect

Based on the MTT assay results, a nontoxic concentration of F_2_BGly (5 μM) was selected. Cells were incubated for 24 h in the dark with F_2_BGly in a culture medium. After this incubation, the cells were washed with PBS and irradiated with a 735 ± 20 nm LED. Next, the cells were washed with PBS, a new portion of fresh medium was added, and the plates were returned to the incubator for 24 h. Cell viability was determined by MTT assay in independent experiments performed 24 h post irradiation. The cell death was examined 24 h post PDT. Fifteen minutes before cytometric measurement, cells were stained with Annexin V-FITC, and then just before the measurement with Propidium iodide (PI) as markers for apoptosis and necrosis cells, respectively. Apoptosis and necrosis were detected with the kit according to the protocol. The measurement was performed using a Guava^®^ easyCyte™ (Merck Millipore, Burlington, MA, USA) flow cytometer equipped with a 488 nm laser. The obtained data were analyzed using InCyte software version 3.2 (Merck Millipore, Burlington, MA, USA).

### 4.11. Intracellular Accumulation

The intracellular accumulation of F_2_BGly was assessed in the CT26 cancer cell line. Prior to imaging, CT26 cells were plated on microscopic slides at a density of 1 × 10^5^ cells and maintained at 37 °C in a humified atmosphere of 95% air and 5% CO₂ atmosphere for 24 h. After being washed with fresh medium, the cells were incubated in the dark with 20 μM solution of F_2_BGly prepared cell medium for 24 h. Cells were stained with Hoechst33342 for 10 min. Following a 30 min incubation at 37 °C in the dark, the cells were washed twice with HBSS, and the slide was placed on the microscope stage. Visualization was performed using a Zeiss LSM 880 confocal microscope (Carl Zeiss, Jena, Germany) equipped with a 63× oil immersion objective. Image analysis was conducted using Zeiss ZEN software version 2.5.

### 4.12. Animal Model

All experiments were carried out with approval no. 190/2018 and 242/2022 of the first and second Ethics Committee for Research on Animals, Krakow, Poland. Eight- to nine-week-old male BALB/c mice (obtained from AnimaLab, Poznań, Poland) were utilized for in vivo PDT studies. The animals were treated ethically and provided with unrestricted access to food and water. The animals were housed and maintained in individually ventilated cages under 50–60% humidity, a 12/12 h light/dark cycle, and at 22 ± 2 °C, in SPF conditions at the Faculty of Biochemistry, Biophysics, and Biotechnology, Jagiellonian University in Krakow, Poland.

### 4.13. Real-Time Whole-Body Imaging

When the tumors reached ca. 0.5 cm in diameter, the mice were injected intravenously with F_2_BGly with a dose of 1.5 mg/kg body weight, and whole-body imaging using the Newton 7.0 Imaging System (Vilber, Collégien, France) was performed immediately following and up to 48 h after the injection. The mice were anesthetized with cocktail ketamine, 75 mg/kg BW (Bioketan, Vetoquinol Biowet, Poland) and medetomidine, 1 mg/kg BW (Domitor, Orion Pharma, Poland) administered intraperitoneally. Fluorescence images of BALB/c mice were collected using λ_ex_ = 680 and λ_em_ = 740–780 nm to illustrate the specific signal for F_2_BGly. After imaging was completed, mice were awakened by intraperitoneal administration of atipamezole (Antisedan, Orion Corporation, Finland).

### 4.14. PDT In Vivo

Mice were randomly assigned to experimental groups (n = 5–6). The CT26 cells (0.35 × 10^6^ in PBS suspension/100 μL) were implanted subcutaneously into the right thigh of BALB/c mice. F_2_BGly (dose: 1.5 mg/kg BW, in CrEL:EtOH:0.9% NaCl (1:2:98)) was administered intravenously into the tail vain when the tumor attained a diameter of 4–5 mm, which usually took about 7–10 days after inoculation. Tumor irradiation was performed at DLI = 15 min using a laser (Omicron laser model LDM750.300.CWA.L.M equipped with optical fiber model FD/Medlight, Ecublens, Switzerland) at 748 nm, with radiant exposures of 74 J/cm^2^ and a laser power of 130 mW. The illuminated region, with a consistent diameter of 1.3 cm, was maintained throughout the irradiation process. During the experiment, the well-being of animals was monitored (tumor size, weight, behavior). When the tumors exceeded 10 mm in diameter, indicating a loss of local tumor control, the mice were euthanized. Survival curves were estimated using Kaplan–Meier analysis.

### 4.15. Statistical Analysis

Statistical analysis was performed using the STATISTICA software version 13.5 for biostatistics (StatSoft Inc., Tulsa, OK, USA). The results are presented as means ± standard deviation or standard error of the mean (SEM) from a minimum of three independent experiments. The difference between groups was evaluated with Student’s *t*-test and considered significant for *p* < 0.05.

## 5. Conclusions

F_2_BGly emerges as a potent photosensitizer for V-PDT, characterized by its exceptional photostability, optimal absorption in the NIR region, and efficient ROS generation. Its ability to mediate both type I and type II photochemical pathways enables comprehensive tumor cell destruction and vascular damage, significantly enhancing its therapeutic efficacy. The glycine modification plays a dual role, facilitating improved cellular uptake and retention while also contributing to its photochemical stability. These features position F_2_BGly as an advanced photosensitizer with the potential to address challenges in treating hypoxic tumors and drug-resistant cancer cell lines.

Future studies should focus on further refining the drug-to-light interval, optimizing dosing regimens, and evaluating long-term therapeutic outcomes in vivo. Additionally, exploring F_2_BGly’s application in cellular-targeted PDT could expand its clinical utility and provide new insights into overcoming limitations associated with conventional photosensitizers.

## Figures and Tables

**Table 1:** The optical and photophysical properties of F_2_BGly, F_2_BOH, and F_2_PGly determined in ethanol along with the partition coefficients (logP_OW_).

Photosensitizer	Absorption λ_max_/nm; ε/M^−1^ cm^−1^						Fluorescence λ_max_/nm	Φ_F_	logP_ow_
	Soret (B)		Q(1–0)		Q(0–0)				
	B_x_	B_y_	Q_x_	Q_y_	Q_x_	Q_y_			
F_2_PGly	410 -		505 -	540 -	584 -	639; 7980	642, 705	0.037	1.7
F_2_BGly	346; 70,100	373; 76,260	505; 36,420		746; 66,580		764	0.096	1.9
F_2_BOH [17]	-	-	-		743; 70,000		764	0.023	−1.4

## Data Availability

The raw data supporting the conclusions of this article will be made available by the authors on request.

## Supplementary Materials

The supporting information can be downloaded at: https://www.mdpi.com/article/10.3390/ijms252313132/s1.

## Abbreviations

APF	3′-(p-aminophenyl) fluorescein
BW	Body weight
CT26	Murine colon carcinoma cell line derived from a BALB/c mouse
DCM	Dichloromethane
DHE	Dihydroethidium
DLI	Drug-to-light interval
DMSO	Dimethyl sulfoxide
EGFR	Epidermal growth factor receptor
F_2_BGly	5,10,15,20-tetrakis[2,6-difluoro-3-(methoxycarbonylmethylsulfamido)-phenyl] bacteriochlorin
F_2_BMet	5,10,15,20-tetrakis[2,6-difluoro-3-(methylsulfamoyl)-phenyl] bacteriochlorin
F_2_PGly	5,10,15,20-tetrakis[2,6-difluoro-3-(methoxycarbonylmethylsulfamido)-phenyl] porphyrin
FDA	Food and Drug Administration
HCl	Hydrochloric acid
HPF	Hydroxyphenyl fluorescein
MCF-7	Human breast cancer cell line
MDA-MB-231	Triple negative breast cancer cell line
MTT	3-(4,5-dimethylthiazolyl-2)-2,5-diphenyltetrazolium bromide
NMR	Nuclear magnetic resonance
PBS	Phosphate-buffered saline
PDT	Photodynamic therapy
PpIX	Protoporphyrin IX
PS	Photosensitizer
ROS	Reactive oxygen species
SOSG	Singlet Oxygen Sensor Green
V-PDT	Vascular-targeted photodynamic therapy
