# Supplementary material for: One Change, Many Benefits: A Glycine-Modified Bacteriochlorin with NIR Absorption and a Type I Photochemical Mechanism for Versatile Photodynamic Therapy
Source: Int J Mol Sci. 2024 Dec 6;25(23):13132. doi: 10.3390/ijms252313132 (PMC11641832; doi:10.3390/ijms252313132)
Supplement: Supplementary file 1 [file ijms-25-13132-s001.zip › ijms-3346105-supplementary.pdf]

International Journal of *Molecular Sciences*

MDPI

Supporting Information for

# One Change Many Benefits: A Glycine-modified Bacteriochlorin with NIR Absorption and Type-I Photochemical Mechanism for Versatile Photodynamic Therapy

**Mateusz Werłos [1,2], Agata Barzowska-Gogola [1,3], Barbara Pucelik [1,3] , Paweł Repetowski [1,4], Marta Warszyńska [1,4], Janusz M. Dąbrowski [1,*]**

[1] Faculty of Chemistry, Jagiellonian University, 30-387 Kraków, Poland
[2] Selvita S.A., ul. Bobrzyńskiego 14, 30-348 Kraków, Poland
[3] Łukasiewicz Research Network, Kraków Institute of Technology, Kraków 30-418, Poland
[4] Doctoral School of Exact and Natural Sciences, Jagiellonian University, 30-348 Kraków, Poland
* Correspondence: jdabrows@chemia.uj.edu.pl; Tel.: +48126682464

## 1. *Photostability*

Photostability was assessed through continuous photolysis. A 5 μM solution of the photosensitizer in PBS (0.5% DMSO) was prepared, and initial absorption spectra were recorded. After irradiation, the spectra were recorded again using a Tecan Infinite M200 Pro plate reader.

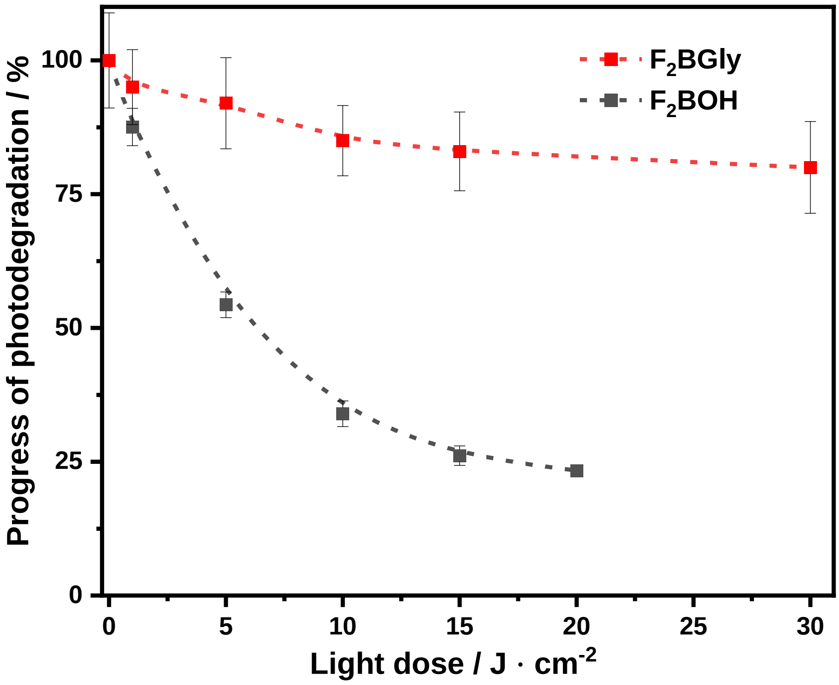


**Figure S1.1** Kinetic curve showing the photodegradation process of $F_2BGly$ in PBS.

International Journal of *Molecular Sciences*

*2.Fluorescence lifetime measurements*

Fluorescence lifetimes were recorded using a Time-Correlated Single Photon Counting (TCSPC) mode using FluoroLog-3 Spectrophotometer (Horiba Jobin Yvon). The instrument was equipped with a 340 nm ps pulsed LED as the excitation source in the MCS mode. During measurements, the Instrument Response Function (IRF) was obtained from a non-fluorescence suspension of colloidal silica (LUDOX 30%, Sigma Aldrich) in water, held in 10 mm path length quartz cell, and was considered to be wavelength independent. All lifetimes were fit to a $\chi_2$ value of less than 1.1 and with residuals trace symmetrically distributed around the zero axes.

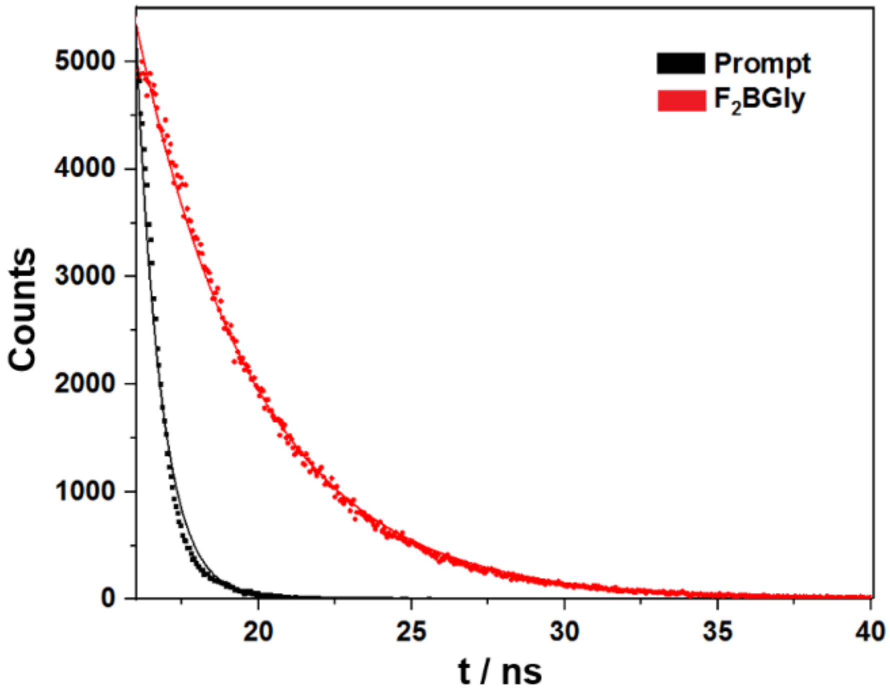


**Figure S2.** Fluorescence decay curves over time for a solution of $F_2BGly$.

International Journal of
*Molecular Sciences*

MDPI

*3. Detection of reactive oxygen species (ROS) with fluorescent probes*

The 3′-p-(aminophenyl)fluorescein (APF) and hydroxyphenyl fluorescein (HPF) are selective probes for hydroxyl radicals. Singlet Oxygen Sensor Green® (SOSG) is a specific probe for singlet oxygen. These probes were employed for the detection of ROS after illumination of the PS. PS solutions were diluted to a final concentration of 10 µM per well. Next, each fluorescent probe was added to a well at a final concentration of 15 µM. PS solutions were irradiated with a 400 ± 20 nm LED light for various light doses. A microplate reader (Tecan Infinite M200 Reader) was used to measure the fluorescence intensity signal immediately before and after illumination with the appropriate excitation and emission parameters.

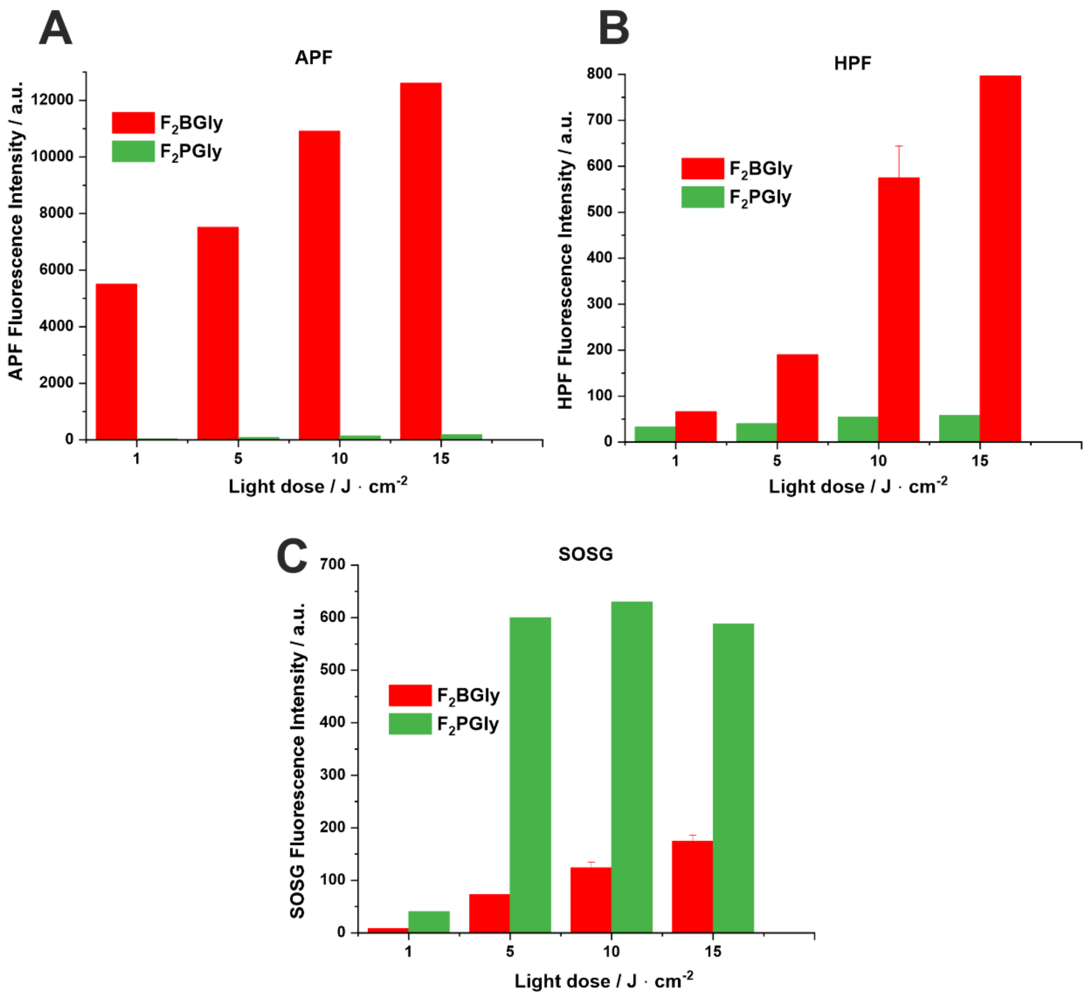


**Figure S2** Green fluorescence of ROS probes– (a) APF, (b) HPF, and (c) SOSG after irradiation of 5 µM aqueous solution (PBS, 0.5% DMSO) of $F_2PGly$ and $F_2BGly$ with light 635 ± 20 nm for porphyrin and 735 ± 20 nm for bacteriochlorin. Results are the average of at least three experiments ± SEM.

*4. Cytotoxicity and cell survival assay*

The MTT (3-(4,5- dimethylthiazol-2-yl)2,5-diphenyl tetrazolium bromide) was used to quantify cell survival and $F_2$PGly-mediated cytotoxicity. After cell attachment to the 96-well plate, $F_2$PGly in a growth medium at concentrations from 0 to 100 μM was added to the cells. Treated cells were incubated for 24h in the dark. Next, the $F_2$PGly solution from each well was removed, cells were washed in PBS and fresh culture medium supplemented with FBS and antibiotics was added to each well, and cells were returned to the incubator for 24h. MTT dissolved in PBS at a content 10% of the final solution was added to each well and the microplates were further incubated for ca. 3h. In the case of MTT, the medium was then discarded and 100 μL of a mixture of DMSO/ methanol (1:1) was added to the cultures and mixed thoroughly to dissolve the dark blue crystals of formazan. Formazan quantification was performed using an automatic microplate reader (Tecan Infinite M200 Reader) by absorbance measurements with a 570 nm test wavelength.

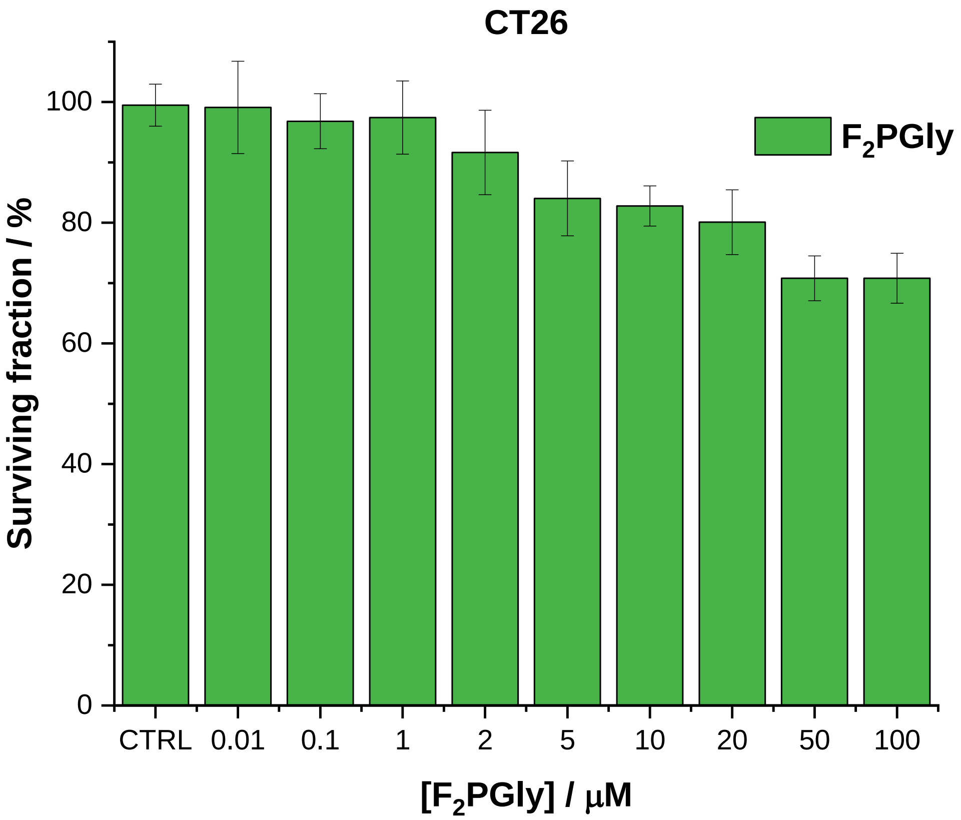


**Figure S3.** Cytotoxicity against CT26 cancer cell line without the irradiation of $F_2$PGly in the concentration range (0,01–100 μM). Results are the average of at least three experiments ± SEM.

*5. Cellular uptake*

CT-26 cells were seeded on 96-plate microplate ($10^4$ per well). After 24h, the cells were incubated with 5 μM $F_2$PGly for time intervals from 2h up to 24h. $F_2$PGly solutions were prepared by diluting its stock solution in DMSO with the culture medium to the desired final concentration (5 μM). The highest concentration of DMSO in the cell growth medium did not exceed 0.5%. After incubation, the cells were washed twice with PBS and solubilized in 30 μL of Triton X-100 and 70 μL of DMSO/ethanol solution (1:3). The retention of cell-associated $F_2$PGly was detected by fluorescence ($\lambda_{exc}$ = 405, $\lambda_{em}$ = 720 nm) with the microplate reader (Tecan Infinite M200 Reader).

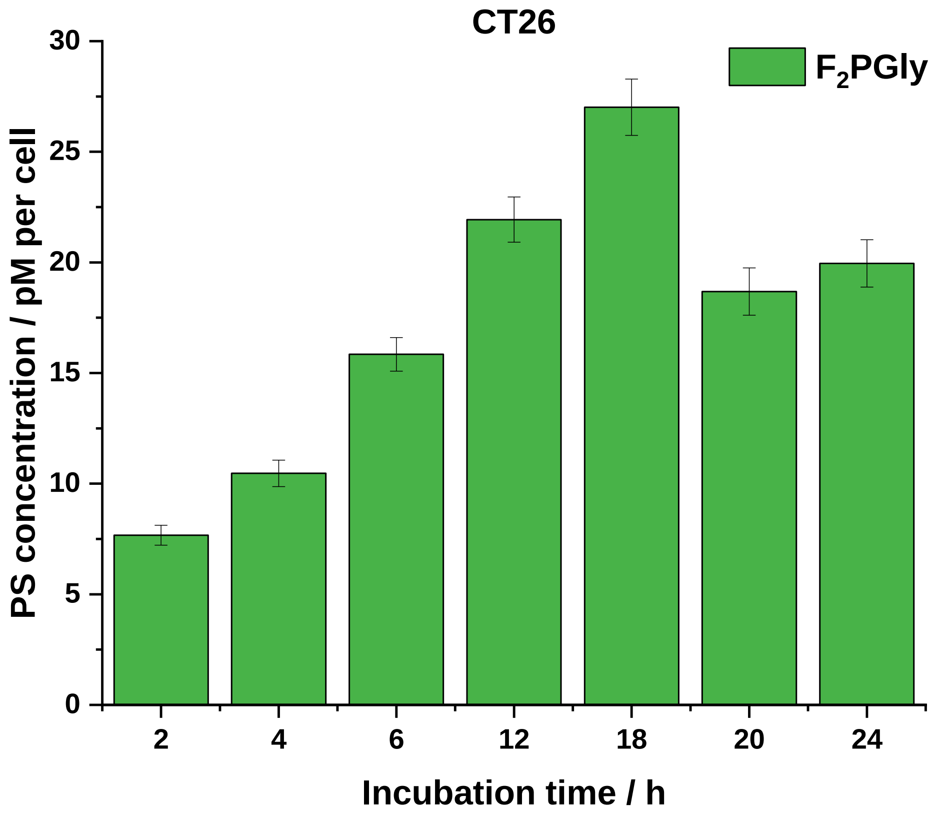


**Figure S4**. Time-dependent uptake of $F_2PGly$ prepared in PBS/0.5% DMSO by CT26 cancer cells measured by fluorescence intensity. Results are the average of at least three experiments ± SEM.

*6. Photodynamic Effect*

On the basis of cytotoxicity results, a nontoxic concentration of $F_2PGly$ (20 μM) was selected. Cells were incubated for 24h in the dark with $F_2PGly$ in a culture medium. After this incubation, the cells were washed with PBS and irradiated with a 635 ± 20 nm LED. Next, the cells were washed with PBS, a new portion of fresh medium was added and the plates were returned to the incubator for 24h. Cell viability was determined by MTT assay in independent experiments performed 24h post-irradiation. The cell death was examined 24 h post-PDT.

International Journal of *Molecular Sciences*

MDPI

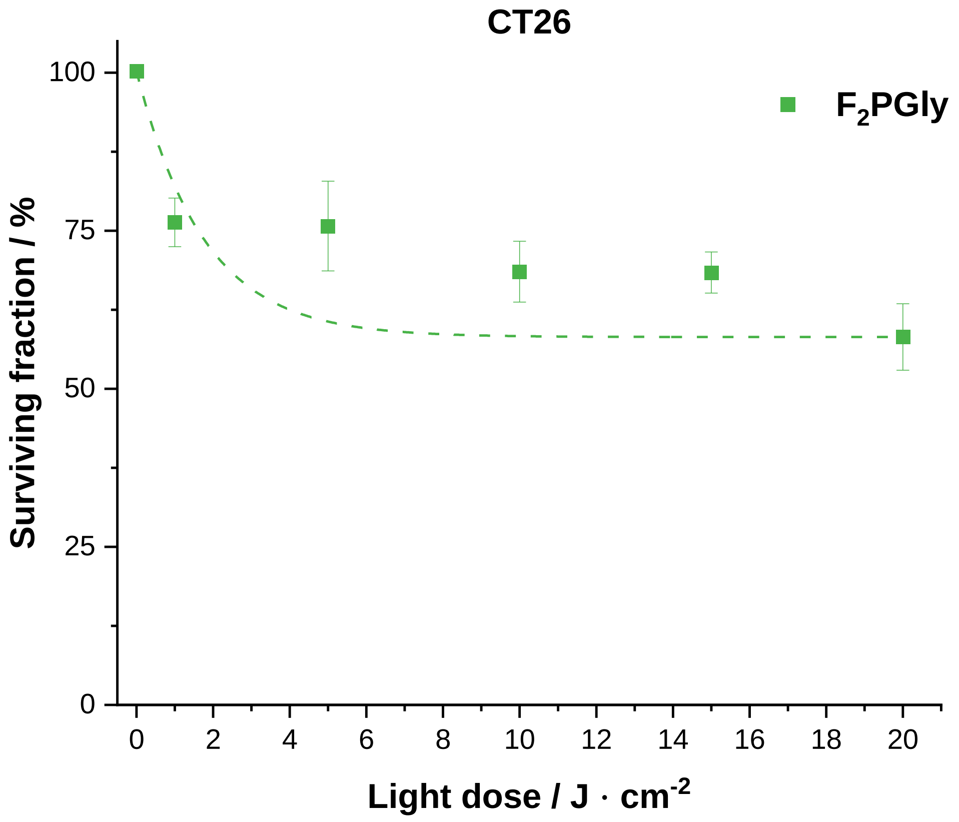


**Figure S5.** Photodynamic effect against CT26 cancer cell line of $F_2$PGly performed after 24h incubation with 20 μM photosensitizer solution (PBS, 0.5% DMSO) and irradiation with red light (635 ± 20 nm).
